# Control of mandibular incisors with the combined Herbst and completely customized lingual appliance - a pilot study

**DOI:** 10.1186/1746-160X-6-3

**Published:** 2010-03-11

**Authors:** Dirk Wiechmann, Rainer Schwestka-Polly, Hans Pancherz, Ariane Hohoff

**Affiliations:** 1 Private Practice, Bad Essen, Germany; 2 Department of Orthodontics, Medizinische Hochschule Hannover, Germany; 3 Department of Orthodontics, University of Giessen, Germany; 4 Department of Orthodontics, Westfälische Wilhelms-Universität Münster, Germany

## Abstract

**Background:**

The traditional Herbst appliance induces mandibular incisor proclination independent of the anchorage system used. The dental effects of the Herbst appliance as an element of a completely customized lingual orthodontic (LO) appliance (Incognito, 3 M) has not been analyzed yet and the aim of this paper was to measure the effect of mandibular incisor proclination using this Herbst-LO device.

**Methods:**

Retrospective study. Inclusion criteria: a) Class II ≥ 5 mm molar relationship; b) Herbst appliance ≥ 9 months in situ; and c) finished active treatment. Incisor position was measured on digital models before treatment, on the digital target setup, and on digital models obtained at the day of debonding. All measurements were performed by one investigator.

**Results:**

Twelve patients (8 females, 4 males) out of 632 cases treated with a lingual appliance were included in the study. The measurement error computed with Dahlberg's formula was 0.2°. Seven cases had planned (target setup) mandibular incisor uprighting (ccr), and five cases had proclination (clockwise rotation). There was no statistical difference (p > 0.05) between planned incisor rotations of the target setup and achieved incisor rotations at the day of debonding. The overall mean difference was 2.2° ± 1.0°.

**Conclusions:**

The Incognito-Herbst combination is the first Herbst device with full control over mandibular incisor movement. Using this system, anchorage loss or anchorage gain is independent of the Herbst treatment. It depends only on the planned tooth position of the individual target setup.

## Background

Treatment using Herbst telescopes can be expected to have different effects. For instance, 87% of overjet reduction is due to dento-alveolar effects, where 47% of the effect can be found in the maxilla and 40%, in the mandible [1]. The proclination of the lower incisors, too, adds to this; after six months into treatment, it may amount to a mean 6.6° [2]. This is undesirable insofar as Angle class II associated with mandibular retrognathism is compensated for in many cases by as little as mandibular incisor proclination.

This treatment-induced proclination, called anchorage loss, has been the issue of various modifications to the appliance attempting to minimize it. So far, several kinds of anchorage have been described: premolar anchorage, premolar-molar anchorage, pelott anchorage, labial-lingual anchorage, class III elastics, splint-type anchorage, and acrylic-type with occlusal coverage [3-5]. The splint-type and the acrylic-type anchorage designs were believed to achieve the best outcomes as all anterior teeth are fitted with brackets and full-size archwires in the case of the former, while in the latter, the teeth are set bodily into plastic material.

Weschler and Pancherz, in their research into various kinds of anchorage, point out matter-of-factly, "*Mandibular anchorage loss in Herbst treatment is a reality with which the orthodontist has to live. Against all expectations, the cast splint anchorage was not superior*" [4]. A systematic review of 2007 reveals the same result: anchorage loss cannot be avoided and mean proclinations of 3.2° - 4.5° are observed even with splint-type and acrylic-type designs [6].

It is all the more surprising that the Herbst appliance combined with a customized bracket system was observed not to exhibit this proclination of the anterior teeth and--quite the opposite--to even result in *anchorage gain *amounting to 7° [7]. This isolated case raised the issue as to whether this was a lucky accident or whether the proclination, unavoidable so far, could be managed by a precisely designed combination of slot and full-size archwire. To answer this question, the first cases of treatment with this new, combined device have now been analyzed.

## Methods

632 patients treated with an individually customized lingual bracket system (Incognito, 3 M) were screened to be enrolled into this study The criteria of inclusion were: a) Angle class II ≥ 5 mm molar relationship; b) Herbst appliance in situ for a minimum of nine months; and c) active treatment completed.

For every single treatment, the Incognito system works with a case-individual *prescription*, which is the result of the pretreatment malocclusion and the individual setup, i.e., the desired treatment outcome. As a consequence, all included cases represent their own, individual *prescription*.

That is why, to assess effects on the proclination of the anterior teeth, the pro-/inclination present as part of the setup has to be considered. For this reason anterior tooth inclination was measured pretreatment (t1) on the scanned casts, as per setup (t2), and with completed active treatment (t3).

**Figure 1:**
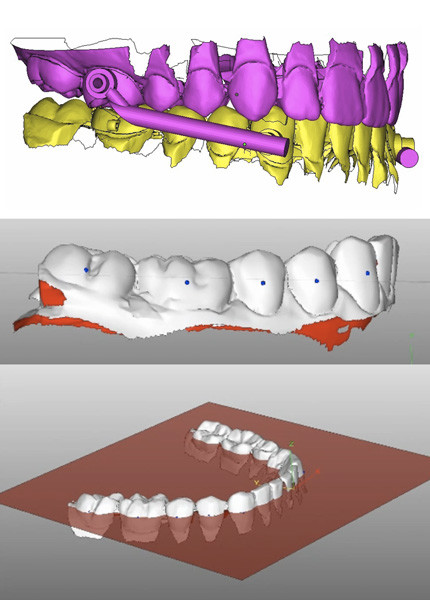
**Top: Planned tooth position of Incognito's setup with virtual Herbst telescopes attached**. Middle: Landmarks placed on the midpoints of the visible crowns (canines, premolars, and molars only) to construct a reproducible reference plane for incisor torque measurements. Bottom: Visual representation of the reference plane.

To establish a reproducible reference plane, landmarks were placed on the midpoints of the visible crowns of canines, premolars, and molars and a horizontal plane was constructed with minimal vertical distance to these landmarks (Figure 1). For measurements the models' postero-anterior plane was oriented to the right-hand side and the most prominent incisor was sectioned parallel to this plane. Incisor torque was measured between the horizontal reference plane and the longitudinal axis of the tooth crown. Incisor reclination was represented as a counter-clockwise (CCW) rotation (negative values) of the tooth axis in the sagittal plane. Incisor proclination was represented as a clockwise rotation (positive values).

To calculate the measurement error one investigator performed replicated incisor measurements on 20 models at two different times. All measurements were taken with the Rhinoceros^® ^NURBS modeler (Robert McNeel & Associates, Woodland Park Avenue North, Seattle WA, U.S.A.). Statistical analysis (descriptive, regression analysis, non-parametric Kruskal-Wallis test) was performed with R [8].

## Results

Based on the inclusion criteria 12 patients (8 females, 4 males) were allocated to the study. At the day of debonding their mean age was 18.6 ± 7.4 years (female: 20.8 ± 8.3 (median 16.2); male: 14.3 ± 1.6 (median 13.6)).

All measurements were performed by one investigator. The measurement error computed with Dahlberg's formula [9] was 0.2°. The maximal range between repeated measurements was 1.2°.

**Figure 2:**
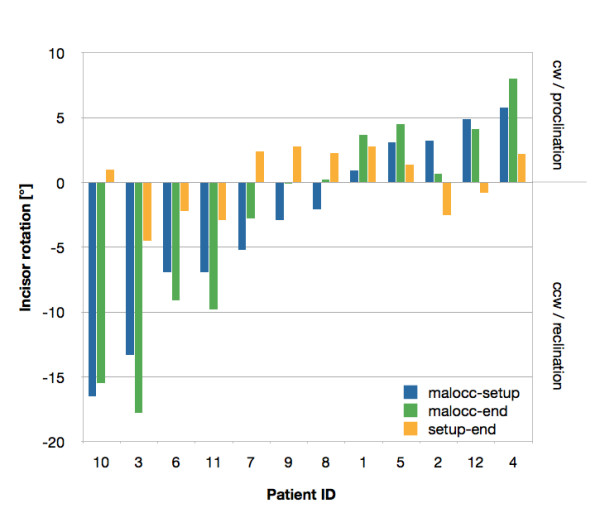
**Distribution of the planned (malocc-setup), the achieved (malocc-end), and the difference between planned and achieved incisor rotations (setup-end)**. Negative values represent a counter-clockwise rotation (reclination), positive values, a clockwise rotation (proclination) of the mandibular incisor in the postero-anterior plane.

Figure 2 shows the distribution of the planned (malocc-setup) and the achieved (malocc-end) mandibular incisor movement. Seven cases were planned with mandibular incisor CCW rotation (mean value = -7.7°) and five cases, with CW rotation (mean value = 3.6°). On the day of debonding all planned incisor movements were achieved except for case 8 where a CCW rotation of -2.1° was planned and a CW rotation of 0.2° was finally measured. The mean difference between planned (malocc-setup) and final incisor rotations (malocc-end) was 2.2° ± 1.0°. However, no statistical difference (p > 0.05) was found between planned and final incisor rotations.

There was a wide range of planned incisor movements, ranging from -16.5° to 5.8°, with two cases (10, 3) with a CCW rotation of more than 13° (Figure 2). For case 10 the planned rotation (-16.5°) was nearly achieved with an undercorrection of 1°, whereas case 3 (13.3° planned) showed an overcorrection of 4.5°. There is no relation between the amount of incisor rotation and the amount of under- or overcorrection (Adjusted R-squared: 0.29). Age has no effect either on the amount of movement (Adjusted R-squared: -0.09).

## Discussion

Up until now there has been an agreement in the literature that anchorage loss in the form of mandibular incisor CW rotation/proclination during Herbst treatment is a reality and cannot be prevented by any anchorage system combined with the Herbst appliance. This paper has been able to show that by using the Incognito's Herbst system, not only could anchorage loss be prevented, but also anchorage gain (in the form of mandibular incisor reclination) could be achieved.

Looking at the anchorage provided by a traditional Herbst appliance, the level of anchorage which a Herbst-LO appliance is able to provide is higher. Its full-arch and full-size, rectangular archwires and the reverse-torque effect on the lower anterior teeth are the reason for this higher level, since the bracket is located posterior to the centre of resistance, which in turn results in a reverse moment of force to which the archwire is exposed. Still higher levels of anchorage can be achieved by using one or a combination of the following: stiffer wires, reverse third-order bends with pre-programmed reverse torque, bracket and pivot bases linked to each other.

The major difference between this study and other retrospective Class II evaluation studies is that only models were used for analysis, instead of lateral head films. Lateral head films would clearly be able to show mandibular incisor inclination before and after treatment, but unable to provide any information on the planned movements. The Incognito's workflow depends on a target plaster cast setup incorporating desired proclination, if any; but if incisor proclination is desired/planned (which was the case 5 times in this study), a mere before-after comparison of head films would have biased the final result because any proclination would have been interpreted as a side effect (anchorage loss) rather than as a planned movement. Therefore, the assessment in this study was based on a reference plane obtained from reproducible landmarks on the digital models. The measurement error of the method was 0.2° and had no effect on the results.

A limiting factor of this study is its retrospective nature and the small number of patients included. The Herbst appliance in Lingual Orthodontics was first described in 2008 [7]. Only a few finished cases are available for analysis as of today and clinically controlled trials do not exist yet. Further investigation based on the presented method is needed to finally verify our preliminary results.

## Conclusions

Considering the retrospective nature of the study and the small number of patients included, the following conclusions could be drawn.

1. The Incognito system allows precise torque control with a mean error of 2.2° ± 1.0° between planned (target setup) and final (day of debonding) tooth position.

2. The Incognito system is able to prevent anchorage loss (mandibular incisor proclination) during Herbst treatment.

3. The Incognito system is able to gain anchorage (mandibular incisor reclination) during Herbst treatment.

4. Using the Incognito system anchorage loss or anchorage gain is independent of the Herbst treatment. It depends only on the planned tooth position in the target setup.

5. The Incognito-Herbst combination is the first Herbst device with full control over mandibular incisor movement.

## Competing interests

DW is the inventor of the Incognito-System and the founder of the former manufacturing company of Incognito which was acquired by 3 M Unitek. DW is working in private practice and not a member or associate of 3 M. All other authors have no competing interests.

## Authors' contributions

HP initiated the investigation, participated in discussions on the undertaking of the study, interpreted the data and reviewed all iterations of the paper. DW and AH designed the study. AH and RS analyzed the data. DW and RS supervised the clinical sample and data collection. DW treated all cases. AH and DW wrote the main part of the paper. RS contributed to writing the paper and reviewed the paper for content. All authors approved the final manuscript.
